# The Story of the Insane from Year to Year

**Published:** 1889-10-05

**Authors:** 


### The Story of the Insane from Year to Year.

### Asylum Reports.

### SUMMARY.

Medical superintendents of asylums have very closely adhered to the tables proposed by the Medico-Psychological Association, and the result is that these statistics are comparatively easy to follow and to understand. It is a great pity that some authorised system should not be adopted in preparing the annual reports. Here there is no order whatever. Some of them are written in the first person, others in the third person, and some skip about from one style to the other. Some are so long that they are spun out to upwards of twenty pages of letterpress, and a few only amount to three pages, or even less. Many of them are carefully arranged into sections, so that there is no difficulty in finding at a glance what is sought for. Others consist of half-a-dozen long paragraphs, as if they were to be read in so many breaths. Of course, it is not an easy thing for a man to have to write on the same subject every year and find something new and original to say on every occasion. No reviewer should expect that, but conciseness and order he may reasonably look for. Some young aspirant for fame might bring this question before the Medico-Psychological Association.

We have followed the precedent of 1888, and have brought under our readers' notice a representative sprinkling of these annual reports, and have done our best to knead them into an harmonious "story of the insane from year to year." Many reports are unavoidably left unnoticed, and that not because they are inferior to the others, but because want of space compels it.

During 1888, the number of the registered insane in England and Wales increased by 1,697, and when the year closed there were known to exist in the country not fewer than 84,340 lunatics. The increase is 55 less than it was in 1887, but it is 287 higher than the average annual increase for the ten preceding years. As usual, the increase has been immensely greater among paupers than among private cases. During the year, 61 criminal lunatics were transferred from Woking Prison to the Broadmoor Asylum, and this raised the total there to 618.

One of the tables in the Blue Book of the Commissioners in Lunacy enables us to compare the numbers of the registered insane on the 1st January, 1859, with the numbers on the 1st January, 1889, and we find that on the former date the proportion of the insane was 18·67 per 10,000 of the population, and on the latter date it had risen to 29·07 per 10,000. We have taken care to point out that these figures refer to the *registered* insane, but making every possible allowance that can be demanded, we fear there is no resisting the conclusion that this terrible disease is increasing among us.

In our short notices of individual asylums we have often given the numbers who were driven insane by drink and of those in whom the disease was owing to inherited influences, and looking at them collectively we see that among the private patients, out of 1,995 admissions 204 owed their insanity to alcoholic intemperance. Among the paupers a still worse tale is told, for out of 12,341 admissions 1,715 found themselves in asylums owing to this frightful evil. Put into percentages, these numbers show that drink accounts for 13·9 per cent. of all the pauper insanity of the country. And these are only the ascertained cases. How many more would have to be added did we but know the truth, it is hard to say. Potent as is alcohol in the causation of insanity, it is dwarfed by hereditary influence. Taking, as before, only ascertained cases, we find that 455 private patients and 2,835 paupers became insane during one year from this cause alone, and drink and hereditary influence taken together actually account for 35 per cent. of the admissions into our asylums in any given year. When we reflect that both are entirely preventable causes, we are lost in amazement at the callousness and thoughtlessness of the people.

One of the most interesting tables in the Blue Book is that which gives the professions of those lunatics admitted during the year 1887. It would seem that every year one actor in 460 becomes insane. This proportion is very high, in fact, it is one of the highest, if not the highest, in the table. Medical men tread close on the heels of the actors, for we learn that their annual contribution to insanity is one in every 567 of their number. It is rather curious to see that every 619 Church of England clergymen supply one lunatic, while it needs 1,349 Dissenting ministers and Catholic priests to provide the unit. The latter calculation is, however, a little disturbed, because scripture-readers and others are included with the ministers and priests. One hotel servant becomes insane out of 595, and only one domestic servant in 1,095. Milliners are not so liable to insanity as their long hours and low wages would lead one to suppose, for we find that they only add to the insane population in the proportion of one in 1,130. A very marked difference exists between the miners and the agricultural labourers, and, contrary to what might be expected, those who burrow in the earth enjoy a greater immunity than those who till the soil. The miner's annual chance of insanity is one in 2,438, while with his agricultural brother it rises to one in 1,533.

On the question of "restraint," that is "mechanical restraint" of the insane, the Commissioners have the following very sensible remarks: "There is an almost universal concensus of opinion in this country that such restraint should be used very sparingly and only under proper restrictions and conditions," and they proceed to say they "could not condemn its employment in every case and without exception, for to do so would be adverse to the interests of the insane themselves. There would always, we considered, be some cases where restraint was necessary, and a mild form of mechanical restraint, such as gloves, sleeves, or the side arm dress was sometimes, we thought, preferable to and less irritating than manual restraint."

It would seem that nearly 14,000 lunatics are confined in workhouses. This number is vastly too high for the comfort and welfare of these cases. It is well nigh impossible that they can be properly looked after in the great majority of workhouses; and as the rate of maintenance in county asylums now shows an average of 8s. 6d. a week, the pecuniary advantage resulting from their detention is very slight; in some counties so slight as to be inappreciable.

With the passing into law of the County Government Bill of 1888, passed also from the hands of the county magistrates the administration of our county asylums, which for so many years they had managed with ability, kindness, and economy. Their duty to the patients and to the ratepayers was almost invariably performed in a right spirit, and whatever may happen under the new system, the insane and the public alike owe a debt of gratitude to the visiting justices, a debt which it is no exaggeration to say will not be easily paid.

